# Bronchoscopy masks mitigate aerosols during upper gastrointestinal endoscopies

**DOI:** 10.1055/a-2173-1711

**Published:** 2023-10-17

**Authors:** Frank Phillips, Jane Crowley, Samantha Warburton, Adolfo Parra-Blanco, George S. D. Gordon

**Affiliations:** 1 574111 NIHR Nottingham Biomedical Research Centre, Nottingham University Hospitals NHS Trust and University of Nottingham, Nottingham, United Kingdom; 2 6123 Department of Electrical and Electronic Engineering, University of Nottingham, Nottingham, United Kingdom of Great Britain and Northern Ireland

**Keywords:** Quality and logistical aspects, Hygiene, Endoscopy Upper GI Tract, Quality management

## Abstract

**Background and study aims** Upper gastrointestinal endoscopies are considered aerosol-generating procedures (AGP) that risk spread of airborne diseases such as SARS-CoV-2. We aimed to investigate where clinically approved bronchoscopy masks applied to patients during esophagogastroduodenoscopies can mitigate spread of aerosols and droplets.

**Patients and methods** This study included patients undergoing routine upper gastrointestinal endoscopy in a standard endoscopy room and used a particle counter to measure size and number of particles 10 cm from the mouths of 49 patients undergoing upper gastrointestinal endoscopies, of whom 12 wore bronchoscopy masks and 37 did not (controls). Particle counts in the aerosol (≤ 5 µm diameter) and droplet (> 5 µm-diameter) size ranges were measured and averaged over the duration of procedures.

**Results** The use of bronchoscopy masks offers a 47% reduction ( *P* = 0.01) in particle count for particles < 5 μm in diameter over the procedure duration (aerosols).

**Conclusions** Bronchoscopy masks or similar are a simple, low-cost mitigation technique that can be used during outbreaks of respiratory diseases such as COVID-19 to improve safety and reduce fallow times.

## Introduction

**Fig. 1:**
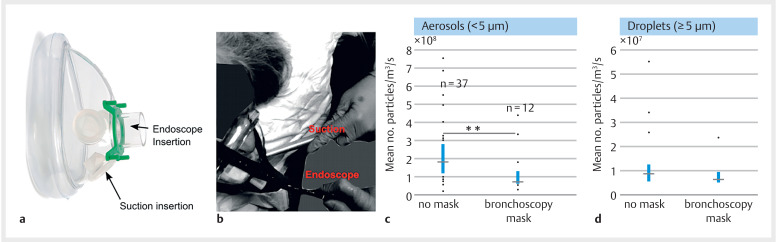
Effect of mitigations on aerosol count. **a** Photograph from procedure showing application of bronchoscopy mask to patient. **b** Effect of bronchoscopy masks, showing significant reduction when being used in the < 5-µm diameter range. * *P* < 0.05, ** *P* < 0.01.

Digestive endoscopy has been proven to produce aerosols 1 2 3 . This represents a risk of infection by COVID-19 and other airborne viruses. The World Health Organization (WHO) has defined aerosols as particles < 5 μm in diameter, which can remain airborne for many hours and can deposit in the lower airways to cause infection, and droplets as particles 5 to 10 μm in diameter, which quickly settle due to gravity but may contaminate surfaces 4 5 . A number of protective barriers have been proposed to minimize that risk. Continuous suction of the oral cavity 1 , shielding barriers 7 8 , masks 9 10 , and increasing the distance between patient and endoscopist 11 and improved ventilation such as laminar flow theaters 12 have been proposed as methods to reduce the exposure of endoscopists and endoscopy staff to aerosols. Here, we present a study that uses masks that are clinically approved for bronchoscopy (Explorer endoscopy facemask, Intersurgical Ltd., United Kingdom) to attenuate aerosol production at the patient’s mouth (bare mask shown in Fig. 1 **a** and in use during an upper gastrointestinal endoscopy in Fig. 1 **b** ). We find that this approach offers 47% ( *P* = 0.01) reduction in particle count for particles < 5 μm in diameter (i.e. aerosols), which are known to spread SARS-CoV-2.

## Materials and methods

To establish the effect of the masks, we measured 12 upper gastrointestinal endoscopy procedures in which the masks were placed on patients immediately before administering xylocaine anesthetic throat spray and were removed after final oral extubation. As a control we measured 37 procedures using normal clinical protocols. This was a prospective study: We randomly selected patients to wear or not wear the protective mask and the outcome of interest was the amount of aerosol produced from the patient’s mouth. A priori power calculations based on limited previous studies determined that with five replicates per patient, we can detect an effect size (Cohen’s d) of 1.98, sufficient to differentiate between a cough and sneeze 13 14 . We also conducted a retrospective power analysis to indicate sample sizes that may be required for further studies.

For both patient groups, procedures were performed across several endoscopy rooms within the same endoscopy suite (Treatment Centre, Nottingham University Hospitals NHS Trust), all of which had similar room ventilation at 15 to 17 air changes per hour (measured using a balometer), similar size, air temperature, and humidity levels. Particle counts were measured and analyzed using an AeroTrak portable particle counter (TSI, Shoreview Minnesota, United States, model 9500–01) with inlet tube placed 10 cm from the patient’s mouth (methodology described in 3 ) to maximize detection sensitivity and for compatibility with previous studies 1 . This device measures particles in six diameter ranges (0.5–0.7 μm, 0.7–1.0 μm, 1.0–3.0 μm, 3.0–5.0 μm, 5.0–10.0 μm, 10.0–25 μm), but for simplicity of analysis, we grouped particles into aerosol and droplet size ranges as defined by the WHO and used in previous studies 3 5 . For greater detail about spatial dispersal around the room, several particle counters could be run simultaneously in different locations as previous studies have done 15 , although care must be taken to avoid reduction in instrument sensitivity because distance from the particle source is increase 16 . All personnel present in the room wore enhanced personal protective equipment (PPE), which minimized the contribution of additional human aerosol sources. Staff and patients were asked to remain as still as possible during recording to avoid scattering of dust or re-aerosolization of liquids on surfaces that might cause increased particle counts. Any unavoidable major movements of people were recorded and time-stamped so they could be excluded if necessary. Previous studies have shown that when particles are measured in this way, there is no significant contribution from events such as biopsy, insertion/removal of catheters, insufflation or diathermy cutting 3 . Therefore, we do not expect that such events would have significantly impacted the results here.

We compared aerosol and droplet concentrations produced from whole procedures (median duration of 7.2 minutes), but we normalized counts to a 20-minute procedure by multiplying total particle count by the appropriate ratio. All statistical analysis was performed using the MATLAB software package (The MathWorks Inc., Massachusetts, United States). Building on existing models of aerosol production in the respiratory tract, we used a log-normal distribution to model the distribution of total particle counts 17 . For the whole-procedure data, a logarithm of the data was first computed, then a t-test was applied to compute *P* values. For individual events, we compared particle counts in 30-second windows before and after an annotated event (e.g. intubation) as per our previously published methodology 3 . For events, the data distribution was modeled as the sum of a log-normal and normal distribution to account for negative values of particle counts that can arise from the subtraction step. A Monte-Carlo sampling method, therefore, was used to provide numerical estimates of *P* value and numerically estimate mean ratios and confidence intervals between events 18 .

## Results

Health Research Authority and ethical approval was granted by the Wales Research Ethics Committee prior to the start of the study (IRAS no. 285595). We included patients undergoing routine upper gastrointestinal endoscopy on the lists of 13 different participating endoscopists at the Endoscopy Unit of the Nottingham University Hospitals NHS Trust Treatment Centre between October 2020 and March 2021. The inclusion criteria were adult patients > 18 years with capacity to consent. Biographical data from the patients is shown in Table 1 . We found that over the period when the bronchoscopy mask was attached, the total number of aerosol-sized particles produced was reduced by 47% (95% CI: 16.8%-65.6%, *P* < 0.01) compared to without masks Fig. 1 **c** . We did not find a significant reduction in total particle count for the droplet range (≥ 5 µm). Considering individual events, we found that the key aerosol-generating events of coughing, extubation, and anesthetic throat spray application were not significantly reduced when the masks were used.

**Table:** **Table 1** Summary table showing demographic data for patients enrolled in this study.

Mask scenario variable	No mask on patient	Bronchoscopy mask on patient
*n*	37	12
Age	Range: 24–93 Median: 61	Range: 41–83 Median: 75.5
Sex	Male: 23, Female: 14	Range: 17-38 Median: 26.9
BMI	Range: 16.3–38.2 Median: 24.8	Range: 17–38 Median: 26.9
Smoking	Smoker: 10 Non-smoker: 27	Smoker: 1 Non-smoker: 10 Vaper: 1
Hiatal hernia	Yes: 10, No: 27	Yes: 5, No: 7
Sedation	Midazolam: 16 Throat spray only: 21	Midazolam: 5 Throat spray only: 7
BMI, body mass index.		

## Discussion

Based on our whole-procedure analysis, we recommend that bronchoscopy masks or similar be used to mitigate aerosols during outbreaks of respiratory diseases such as COVID-19. However, our analysis of individual events suggests that although the masks are effective at containing continuous low-volume aerosols production, e.g. breathing, they are less effective at containing fast, high-volume production events. We suggest that this is due to the openings in the mask required for breathing and the relatively constant rate of suctioning: If aerosol production events exceed the suction rate, these particles will necessarily escape via these holes. Further reduction for individual events may require the use of negative pressure masks [18].

There are a number of limitations and remaining questions following this study. First, particles greater than 5 µm in diameter (droplets) do not appear to be greatly impacted by the masks. This may because they are relatively low in number and that a larger sample size is needed to see this effect. Our retrospective analysis of study power suggests that given the measured data in the aerosol size range with our sample size of n = 12, study power is 0.88, which is comfortably above the threshold of 0.8 usually required for such studies. However, larger studies in the future may be required to better predict, understand, and mitigate flow, distribution, and elimination behaviors of aerosols and droplets. Further, it will also be important to measure a wider range of procedures and to use particle counters in different room positions. This may help to examine the impact of other sources of aerosols in the rooms and to examine the impact of events such as biopsies, diathermy cutting, and insertion/removal of catheters, although our previous work did not find these to be significant producers of particle when measured near a patient’s mouth 3 . Finally, although the endoscopists did not note significant reduction in maneuverability of the endoscope due to the presence of the mask, future studies should evaluate this thoroughly by performing post-procedure surveys of different endoscopists.

## Conclusions

Overall, the reduction in particle levels may be sufficient to warrant reduced fallow time because fewer particles mean shorter air clearance time, but is not sufficient to eliminate the need for PPE for healthcare staff. We recommend that improved masks be designed that can mitigate aerosols more effectively.
